# A Gene of the β3-Glycosyltransferase Family Encodes *N*-Acetylglucosaminyltransferase II Function in Trypanosoma brucei*

**DOI:** 10.1074/jbc.M116.733246

**Published:** 2016-05-04

**Authors:** Manuela Damerow, Frauke Graalfs, M. Lucia S. Güther, Angela Mehlert, Luis Izquierdo, Michael A. J. Ferguson

**Affiliations:** From the Division of Biological Chemistry and Drug Discovery, School of Life Sciences, University of Dundee, Dundee DD1 5EH, Scotland, United Kingdom

**Keywords:** glycobiology, glycosyltransferase, parasite, post-translational modification (PTM), Trypanosoma brucei, N-acetylglucosamine

## Abstract

The bloodstream form of the human pathogen *Trypanosoma brucei* expresses oligomannose, paucimannose, and complex *N*-linked glycans, including some exceptionally large poly-*N*-acetyllactosamine-containing structures. Despite the presence of complex *N*-glycans in this organism, no homologues of the canonical *N*-acetylglucosaminyltransferase I or II genes can be found in the *T. brucei* genome. These genes encode the activities that initiate the elaboration of the Manα1–3 and Manα1–6 arms, respectively, of the conserved trimannosyl-*N*-acetylchitobiosyl core of *N*-linked glycans. Previously, we identified a highly divergent *T. brucei N*-acetylglucosaminyltransferase I (TbGnTI) among a set of putative *T. brucei* glycosyltransferase genes belonging to the β3-glycosyltransferase superfamily (Damerow, M., Rodrigues, J. A., Wu, D., Güther, M. L., Mehlert, A., and Ferguson, M. A. (2014) *J. Biol. Chem.* 289, 9328–9339). Here, we demonstrate that TbGT15, another member of the same β3-glycosyltransferase family, encodes an equally divergent *N*-acetylglucosaminyltransferase II (TbGnTII) activity. In contrast to multicellular organisms, where GnTII activity is essential, *TbGnTII* null mutants of *T. brucei* grow in culture and are still infectious to animals. Characterization of the large poly-*N*-acetyllactosamine containing *N*-glycans of the *TbGnTII* null mutants by methylation linkage analysis suggests that, in wild-type parasites, the Manα1–6 arm of the conserved trimannosyl core may carry predominantly linear poly-*N*-acetyllactosamine chains, whereas the Manα1–3 arm may carry predominantly branched poly-*N*-acetyllactosamine chains. These results provide further detail on the structure and biosynthesis of complex *N*-glycans in an important human pathogen and provide a second example of the adaptation by trypanosomes of β3-glycosyltransferase family members to catalyze β1–2 glycosidic linkages.

## Introduction

The African trypanosomes are protozoan parasites that cause human African sleeping sickness and Nagana in cattle. The parasite undergoes a complex life cycle between the mammalian host and the blood-feeding tsetse fly vector (*Glossina* sp.). Throughout this life cycle, *Trypanosoma brucei* is coated by glycosylphosphatidylinositol (GPI)^8^-anchored proteins. The bloodstream form of the parasite in the mammalian host is covered by a coat of 5 × 10^6^ variant surface glycoprotein (VSGs) homodimers and evades the immune system by replacing one VSG coat by another, in a process known as antigenic variation (1–4). The VSG GPI anchors contain side chains of 0–6 Gal residues, depending on the VSG variant (5–7) and between 1 and 3 *N*-linked glycans. The latter can be of oligomannose, paucimannose, or complex types (6, 8, 9). *T. brucei* expresses numerous other GPI-anchored and transmembrane glycoproteins at the cell surface, in the flagellar pocket, and in the intracellular endosomal/lysosomal system, some of which are life cycle stage-specific or display life cycle stage-specific glycosylation differences. For example, the transmembrane invariant surface glycoproteins ISG65 and ISG75 (10) and the GPI-anchored flagellar pocket ESAG6/ESAG7 heterodimeric transferrin receptors (11–13) are specific to the bloodstream life cycle stage, whereas the major lysosomal glycoprotein p67 is common to bloodstream and procyclic stages but contains complex *N*-glycans only in the bloodstream stage (14). This control of stage-specific glycosylation resides primarily at the level of oligosaccharyltransferase expression (15). Thus, in the bloodstream form of *T. brucei* both the *TbSTT3A* and *TbSTT3B* genes are expressed, and it appears that TbSTT3A co-translationally scans for glycosylation sequons in relatively acidic local environments, transferring exclusively Man_5_GlcNAc_2_ that is destined to be processed to paucimannose or complex *N*-glycans, whereas TbSTT3B post-translationally modifies any remaining sequons with Man_9_GlcNAc_2_ that is destined to be processed no further than Man_5_GlcNAc_2_ in the conventional oligomannose series. Conversion from the oligomannose series to the complex series by the conventional mammalian-type route cannot occur because the parasite lacks a Golgi α-mannosidase II gene (16). In the procyclic form of *T. brucei,* the expression of *TbSTT3A* is repressed at both the mRNA level (15) and protein level (17), favoring the transfer of Man_9_GlcNAc_2_ and the predominant expression of the conventional Man_5_GlcNAc_2-_Man_9_GlcNAc_2_ oligomannose series (18).

The survival strategies of protozoan parasites frequently involve the participation of glycoconjugates. *T. brucei* expresses many glycoproteins containing Gal and GlcNAc, including glycoproteins with novel bloodstream form-specific giant poly-*N*-acetyllactosamine (poly-LacNAc) containing *N*-linked glycans (19). The creation of UDP-glucose 4′-epimerase (*TbGalE*) conditional null mutants showed that this gene, and hence UDP-Gal, is essential for the survival of the parasite in both the bloodstream and procyclic form life stages (20–22). Similarly, the creation of UDP-GlcNAc pyrophosphorylase (*TbUAP*) and glucosamine 6-phosphate *N*-acetyltransferase (*TbGNA*) conditional null mutants has shown that UDP-GlcNAc is essential for bloodstream form of *T. brucei* (23, 24). From these experiments, it is possible to conclude that one or more of the UDP-Gal- and UDP-GlcNAc-dependent glycosylation pathways are essential to the parasite. This has provided the impetus to identify and characterize the UDP-Gal- and UDP-GlcNAc-dependent glycosyltransferase (GT) genes in the parasite. We previously reported a family of 21 genes with predicted amino acid sequences consistent with being UDP-sugar-dependent GTs. All 21 putative *T. brucei* GT amino acid sequences are similar to those of the mammalian β3GT family (25). The mammalian β3GT family includes Gal, Glc, glucuronic acid, GlcNAc, and GalNAc β-3 transferases, and its members contain N-terminal transmembrane domains followed by three conserved motifs as follows: (I/L)R*XX*WG, (F/Y)(V/L/M)*XXX*D*X*D, and (E/D)D(A/V)(Y/F)*X*G*X*(C/S). The comparable motifs in the *T. brucei* genes are slightly different, WG, Y(I,V,F)*X*K*X*DDD, and ED(A/V/I/L/M)(M/L)*X*(G/A), but nevertheless, they identify the parasite genes as belonging to the β3GT superfamily (26). One of these genes (*TbGT8*) encodes a β1–3 GlcNAc transferase and another (*TbGT3*) a β1–3 Gal transferase that modifies the complex GPI anchor side chains of the procyclins (the major surface glycoproteins of the procyclic life cycle stage) (26–28). However, we recently reported that another gene (*TbGT11*) encodes a β1–2 GlcNAc transferase that performs a similar role to members of the *N*-acetylglucosaminyltransferase I family, in that it transfers GlcNAc in β1–2 linkage to the 3-arm of Manα1–6(Manα1–3)Manβ1-4GlcNAcβ1–4GlcNAc (1).

Here, we report that another *T. brucei* β3GT superfamily gene member (*TbGT15*) encodes another β1–2 GlcNAc transferase that was already localized to the Golgi apparatus (29, 30). It performs a similar role to members of the *N*-acetylglucosaminyltransferase II family in that it transfers GlcNAc in β1–2 linkage to the 6-arm of Manα1–6(Manα1–3)Manβ1–4GlcNAcβ1-4GlcNAc, emphasizing the highly divergent nature of the trypanosome genes involved in structurally conserved aspects of complex *N*-glycan biosynthesis.

## Experimental Procedures

### 

#### 

##### Cultivation of Trypanosomes

*T. brucei brucei* strain 427 bloodstream form parasites, expressing VSG variant 221 and transformed to stably express T7 polymerase and the tetracycline repressor protein under G418 antibiotic selection (31), were used in this study. This genetic background will be referred to as wild-type (WT). Cells were cultivated in HMI-9 medium containing 2.5 μg/ml G418 at 37 °C in a 5% CO_2_ incubator as described previously (31).

##### DNA and RNA Isolation and Manipulation

Plasmid DNA was purified from *Escherichia coli* (α-select chemically competent cells, Bioline, London, UK) using Qiagen Miniprep or Maxiprep kits, as appropriate. Gel extraction and reaction clean up was performed using QIAquick kits (Qiagen). Custom oligonucleotides were obtained from Eurofins MWG Operon or the Dundee University oligonucleotide facility. *T. brucei* genomic DNA was isolated from ∼2 × 10^8^ bloodstream form cells using DNAzol (Helena Biosciences, UK) by using standard methods. *T. brucei* mRNA was extracted from 1 × 10^7^ cells using RNeasy RNA extraction kit (Qiagen).

##### Generation of Gene Replacement Constructs

The 517-bp 5′ and 454-bp 3′ UTR sequences next to the Tb427.7.300 ORF were PCR-amplified from genomic DNA using *Pfu* DNA polymerase with primers 5′-cgttGTCGACagtatccgcaaaatgcgact-3′ and 5′-*gtttaaac*ttacggaccgtcaagctttatttttctttccctacgcac-3′ and 5′-gacggtccgtaag*tttaaac*ggatccaaagcggaataaaaataaatc-3′ and5′-ataagtaaGCGGCCGCagatgtcgcgcaagaaaaac-3′ as forward and reverse primers, respectively. The two PCR products were used together in a further PCR to yield a product containing the 5′-UTR linked to the 3′-UTR by a short HindIII (underlined), PmeI (italics), and BamHI (underlined) cloning site and NotI and SalI restriction sites at each end (capital letters). The product was cloned between the NotI and SalI sites of the pGEM-5Zf(+) vector (Promega).

The hygromycin phosphotransferase (*HYG*) and puromycin acetyltransferase (*PAC*) drug-resistance genes were then introduced into the targeting vector via the HindIII and BamHI cloning sites. For re-expression of Tb427.7.300, the ORF was PCR-amplified from genomic DNA with the primer pair 5′-agaaagcttatggtgtggagtgggcataaa-3′ and 5′-ttcagatcttcatgtgcacgaggcgtgcca-3′ and cloned into pLEW100-Phleo (31).

For overexpression of full-length TbGT15 with a C-terminal 3× HA epitope tag, a plasmid was generated based on the trypanosome expression vector pLEW82 (31). *TbGT15* ORF was amplified from *T. brucei* genomic DNA and the primers 5′-gactaagcttatggtgtggagtgggcataaatac-3′ and 5′-gactttaattaa*tgcgtaatcagggacgtcataaggatatgcgtaatcagggacgtcataaggata*cgctcccgcTGTGCACGAGGCGTGCCATC-3′ containing a HindIII and PacI restriction site (underlined), respectively. The sequence encoding for two HA tags (italics) followed by a sequence encoding an Ala-Gly-Ala linker was attached as a 5′-overhang of the reverse primer. The PCR product was subcloned into pLEW82-*GPIdeAc-HA* (32) via HindIII and PacI restriction sites under replacement of the *GPIdeAc* insert, but retention of the sequence encoding for one HA tag, resulting in the plasmid pLEW82-*TbGT15-HA*_3_. The identity of all constructs was confirmed by sequencing.

##### Transformation of Bloodstream Form T. brucei

Constructs for gene replacement and ectopic expression were purified, digested with NotI to linearize, precipitated, washed with 70% ethanol, and re-dissolved in sterile water. The linearized DNA was electroporated into *T. brucei* bloodstream form cells (strain 427, variant 221) that were stably transformed to express T7 RNA polymerase and the tetracycline repressor protein under G418 selection. Cell culture and transformation were carried out as described previously (31–33).

##### Southern Blotting

Aliquots of genomic DNA isolated from 100 ml of bloodstream form *T. brucei* cultures (∼2 × 10^8^ cells) were digested with EcoRI, resolved on a 0.8% agarose gel and transferred onto a Hybond-N positively charged membrane (GE Healthcare, UK). Highly sensitive DNA probes labeled with digoxigenin-dUTP were generated using the PCR digoxigenin probe synthesis kit (Roche Applied Science) according to the manufacturer's recommendations and hybridized overnight at 42 °C. Detection was performed using alkaline phosphatase-conjugated anti-digoxigenin Fab fragments and the chemiluminescent substrate CSPD (Roche Applied Science).

##### Mouse Infectivity Studies

Wild-type and *TbGT15* null mutant bloodstream form trypanosomes were grown in HMI-9T media, washed in media without antibiotics, and resuspended at 5 × 10^6^ cells/ml. Groups of five female BALB/c mice were used for each cell line, and 0.1 ml of the suspension above was injected intraperitoneally per animal. Infections were assessed 3 days post-infection by tail bleeding and cell counting using a Neubauer chamber in a phase contrast microscope.

##### Semi-quantitative RT-PCR

To assess the amount of Tb427.7.300 mRNA in the *TbGT15* conditional null mutant cells grown under permissive and non-permissive conditions, RT-PCRs were performed using AccessQuick RT-PCR System (Promega) according to the manufacturer's recommendations. A *TbGT15* 350-bp fragment was amplified with the primer pair 5′-cacattgtcgcgggatgtgagtgag-3′ and 5′-ccatcccaagtacccgcggtaaaatggg-3′. As a control to ensure similar RNA levels in both samples, primers 5′-aatggatgcggaccttcagcacccac-3′ and 5′-tagaaccgtgagcgcggtgccatac-3′ amplifying a 448-bp product of dolichol phosphate mannose synthase (Tb10.70.2610) were used.

##### Small Scale sVSG Isolation

Soluble form VSG (sVSG) was isolated from 100 ml of cultures containing ∼2 × 10^8^ bloodstream form *T. brucei* by a modification of the method of Cross and co-workers (34, 35) as described previously (36). Briefly, cells were chilled on ice, centrifuged at 2500 × *g* for 10 min, and washed in an isotonic buffer. The pellet was resuspended in 300 μl of lysis buffer (10 mm NaH_2_PO_4_ buffer, pH 8.0, containing 0.1 mm tosyllysine chloromethyl ketone hydrochloride (TLCK), 1 μg/ml leupeptin, and 1 μg/ml aprotinin) and incubated for 5 min at 37 °C. The sample was centrifuged at 14,000 × *g* for 5 min, and the supernatant was applied to a 200-μl DE52 anion exchange column pre-equilibrated in 10 mm sodium phosphate buffer, pH 8.0. Elution was performed with 0.8 ml of 10 mm sodium phosphate buffer, pH 8.0, and the eluate was concentrated and diafiltered with water on a YM-10 spin concentrator (Microcon). The final sample of 50–100 μg of sVSG221 was recovered in a volume of 100 μl of water.

##### ES-MS Analysis of Intact sVSG

50 μg of aliquots of sVSG preparations were diluted to ∼0.05 μg/μl in 50% methanol, 1% formic acid and analyzed by positive ion ES-MS on a Q-Tof 6520 instrument (Agilent). Data were collected, averaged, and processed using the maximum entropy algorithm of the MassHunter software (Agilent).

##### Lectin Blotting of Cell Extracts

To analyze *N-*glycosylation of *T. brucei* bloodstream form cells, ∼2 × 10^8^ cells were first depleted of VSG by hypotonic lysis (34, 35). For Western blot analysis, residual cell ghosts were solubilized in SDS sample buffer containing 8 m urea, boiled with DTT, separated by SDS-PAGE (∼1 × 10^7^ cell eq/lane) on NuPAGE bis-Tris 4–12% gradient acrylamide gels (Invitrogen) and transferred to a nitrocellulose membrane (Invitrogen). Ponceau S staining confirmed equal loading and transfer. Glycoproteins were probed with 1.7 μg/ml biotin-conjugated ricin (RCA-120, Vector Laboratories, UK) in PBS before or after pre-incubation with 10 mg/ml d-galactose and 10 mg/ml α-lactose to confirm specific ricin binding. Detection was performed using IRDye 680LT-conjugated streptavidin and the LI-COR Odyssey infrared imaging system (LICOR Biosciences, Lincoln, NE).

##### Structural Analysis of the Large N-Glycan Fraction

Bloodstream form cells of wild-type and *TbGT15* null mutant cells were isolated from infected rats and processed as described (19). Briefly, VSG-depleted cell ghosts of 1 × 10^11^ cell eq were solubilized with SDS/urea buffer followed by lectin affinity chromatography using ricin-agarose (RCA-120, Vector Laboratories). *N-*Glycans from the ricin-binding glycoproteins were released with peptide:*N*-glycosidase F (*Flavobacterium meningosepticum*, Roche Applied Science) and applied to a Bio-Gel P-4 gel filtration column. Aliquots of eluted fractions were subjected to methanolysis, trimethylsilylation, and GC-MS monosaccharide composition analysis (37). Fractions that eluted in the void volume of the column (the total poly-LacNAc fraction, rich in Gal and GlcNAc) were pooled and used for methylation linkage analysis. After permethylation, acid hydrolysis, NaBD_4_ reduction, and acetylation, the resulting partially methylated alditol acetates (PMAAs) were analyzed by GC-MS (Agilent) as described previously (38). Authentic glycans of Galβ1–4GlcNAcβ1–2Manα1–6(Galβ1–4GlcNAcβ1–2Manα1–3)Manβ1-4GlcNAcβ1–4GlcNAc, lacto-*N*-neohexaose Galβ1–4GlcNAcβ1–6(Galβ1–4GlcNAcβ1–3)Galβ1–4Glc, lacto-neotetraose Galα1–4Glclβ1–3Galβ1–4GlcNAc, and Galβ1–6Gal (Dextra Laboratories, UK) were subjected to methylation linkage analysis alongside the experimental samples. Using the PMAA derivative derived from non-reducing terminal galactose residues, common to all of these structures (*i.e.* 1,5-di-*O-*acetyl-2,3,4,6-tetra-*O-*methyl-1-[^2^H]galactitol), we were able to inter-relate these data and determine the total ion current molar relative response factors for the PMAAs derived from terminal-Gal, 3-*O-*substituted Gal, 6-*O-*substituted Gal 3,6-di-*O-*substituted Gal, 2-*O-*substituted Man, 3,6-di-*O-*substituted Man, and 4-*O-*substituted GlcNAc (1.59, 0.99, 0.27, 0.90, 1.20, 1.00, and 0.23, respectively). These molar relative response factor values were used to correct the peak integrations of sample PMAA total ion current chromatograms and thus provide molar ratios of the PMAAs in the methylation linkage analyses of the wild-type and TbGT15 null glycan samples.

##### GnTII in Vitro Activity Assay

TbGT15 fused to a C-terminal triple HA tag was overexpressed in *T. brucei* bloodstream form cells. 1 × 10^9^ cells were lysed on ice in 25 mm Tris, pH 7.5, 100 mm NaCl, 1% Triton X-100 containing a mixture of protease inhibitors (CompleteMini, Roche Applied Science), and 0.1 mm TLCK. Expression was confirmed by SDS-PAGE and Western blotting. Briefly, 5 × 10^6^ cell eq/lane were separated on NuPAGE bis-Tris 4–12% gradient acrylamide gels (Invitrogen) and transferred to nitrocellulose membrane (Invitrogen). Ponceau S staining confirmed equal loading and transfer. Detection was performed using 0.5 μg/ml rabbit anti-HA antibody (QED Bioscience Inc., San Diego) and IRDye 680LT-conjugated donkey anti-rabbit IgG and the LI-COR Odyssey infrared imaging system (LICOR Biosciences). For the *in vitro* activity assay, TbGT15-HA_3_ was immunoprecipitated using anti-HA magnetic beads (Pierce) and incubated with 1 μCi of UDP-[^3^H]GlcNAc (specific activity of 20–40 Ci/mmol, PerkinElmer Life Sciences), 1 mm cold UDP-GlcNAc (Sigma), and 5 μg of Manα1–6(Manα1–3)Manβ1–4GlcNAcβ1–4GlcNAc or 25 μg of α1–3,α1–6-mannotriose (both Dextra Laboratories, Reading, UK) in 50 mm Tris, pH 7.5, 10 mm MgCl_2_, 10 mm MnCl_2_ in a total volume of 50 μl. After overnight incubation under vigorous shaking at room temperature, samples were desalted via a mixed-bed ion exchange column of 100 μl of Chelex-100 (Na^+^) over 100 μl of AG50X12 (H^+^) over 200 μl of AG3X4 (OH^−^) over 100 μl of QAE-Sephadex 25 (OH^−^), all from Bio-Rad, UK, except QAE-Sephadex (Sigma). Finally, glycans were freeze-dried and re-dissolved in 20% 1-propanol, and aliquots were spotted onto silica HPTLC plates (SI-60 HPTLC, Millipore) that were run twice in 1-propanol/acetone/H_2_O (9:6:4). For product analysis, samples were treated with 128 units of α1–2,3 mannosidase from *Xanthomonas manihotis* (New England Biolabs) or 0.2 units of β-*N-*acetylglucosaminidase from *Canavalia ensiformis* (Sigma) before TLC analysis. Plates were then dried, sprayed with EN^3^HANCE autofluorography enhancer (EN^3^HANCE, PerkinElmer Life Sciences), and exposed on x-ray film at −80 °C for 1–2 days.

For mass spectrometric analysis of the reaction product, the assay was performed using 5 mm non-radioactive UDP-GlcNAc. Samples were analyzed by LC-MS using a HILIC column (Tosoh TSKgel Amide column, 1 mm × 10 cm) and a gradient of 80 to 5% acetonitrile in 0.1% formic acid at a flow rate of 50 μl/min using a TSQ Quantiva triple-quadrupole mass spectrometer (Thermo Fisher Scientific). For methylation linkage analysis of the product, glycans were converted to constituent monosaccharides in the form of partially methylated alditol acetates and analyzed by GC-MS as described above.

##### Scanning Electron Microscopy

To analyze bloodstream form cells by scanning electron microscopy, cells were fixed in HMI-9 medium with 2.5% glutaraldehyde. They were further processed and examined in a Philips XL30 ESEM operating at an accelerating voltage of 15 kV by the Centre for High Resolution Imaging and Processing (CHIPS) at the University of Dundee.

## Results

### 

#### 

##### Analysis of the TbGT15 Gene Product

We previously characterized the biological function of three members of a family of putative UDP-sugar-dependent GTs (1, 26, 27). In this study, Tb927.7.300 was selected for functional analysis. The gene encodes for a 367-amino acid protein with a theoretical molecular mass of 43.1 kDa. Stable isotope labeling with amino acids in cell culture-based quantitative proteomic data demonstrated that the protein expression level is 15 times higher in bloodstream form parasites compared with procyclic form parasites (40).

The *T. brucei* strain that was used in this study (Lister strain 427) differs from the one that was used for the reference genome sequencing project (TREU927). However, an alignment of Tb927.7.300 and its homologue Tb427.7.300 revealed a very high similarity with only three single nucleotide polymorphisms, none of them resulting in amino acid changes. The strain 427 gene and protein product will be referred to here as *TbGT15* and TbGT15, respectively.

The protein sequence contains several hallmarks of Golgi apparatus glycosyltransferases. First, a membrane protein topology prediction program based on a hidden Markov model (41) designates TbGT15 as a type II transmembrane protein. In addition, the sequence contains a D*X*D motif, which is generally involved in catalytic activity of known GTs (42) as well as a dibasic motif, which functions as an endoplasmic reticulum exit signal (43). Indeed, a subcellular Golgi localization of TbGT15 was confirmed previously (29, 30).

##### Creation of Bloodstream Form TbGT15 Null and Conditional Null Mutants

**FIGURE 1.**
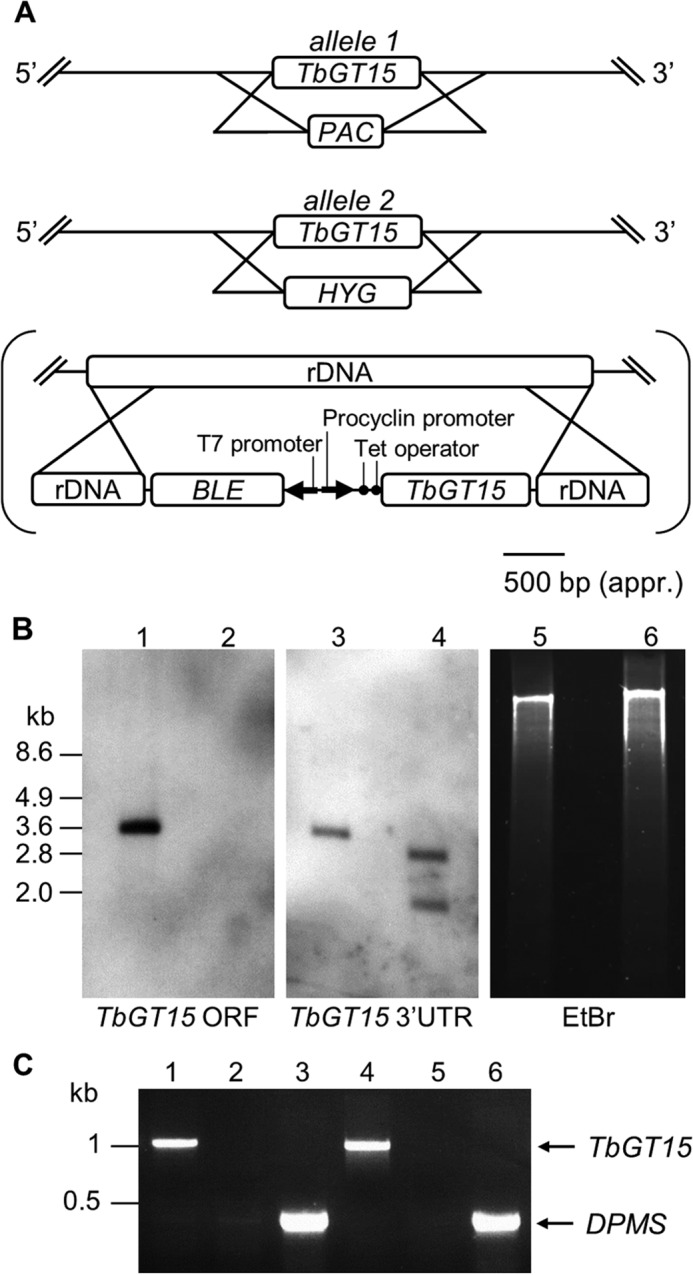
**Generation of a bloodstream form *TbGT15* null and conditional null mutant.** *A*, gene replacement strategy to create *TbGT15* null mutant cells and subsequent insertion of tetracycline-inducible ectopic copy, in *brackets*, to create a conditional null mutant. *B*, Southern blot of genomic DNA digested with EcoRI from WT (*lanes 1, 3,* and *5*) and *TbGT15* null mutant cells (*lanes 2, 4,* and *6*). The blot was probed with a *TbGT15* ORF probe (*left panel*) and a *TbGT15* 3′UTR probe (*middle panel*) and shows the replacement of both alleles with drug resistance cassettes. Equal loading was verified by ethidium bromide staining (*right panel*). *C,* ethidium bromide-stained agarose gel of reverse transcription-PCR products from RNA extracted from WT cell, *TbGT15* null, and conditional null mutants. *TbGT15* mRNA was detected in WT (*lane 1*) and *TbGT15* conditional null mutant cells grown under permissive (plus tetracycline) conditions (*lane 4*), although no mRNA was found in *TbGT15* null mutants (*lane 2*) and *TbGT15* conditional null mutants grown under non-permissive conditions (*lane 5*). To show equal RNA input, a control using dolichol-phosphate mannose synthase (*DPMS*) primers was performed with *TbGT15* null mutants (*lane 3*) and *TbGT15* conditional null mutants grown under non-permissive conditions (*lane 6*).

As TbGT15 is predominantly expressed in bloodstream form parasites (40), we decided to investigate the protein function by creating null and conditional null mutants in this life cycle stage. BLAST search of the *T. brucei* genome suggested that *TbGT15* is present as a single copy per haploid genome. Both alleles were sequentially replaced by homologous recombination using *PAC* and *HYG* drug resistance cassettes as summarized in Fig. 1*A*. After selection on the respective antibiotics, the generation of a *TbGT15* null mutant (Δ*TbGT15*::*PAC*/Δ*TbGT15*::*HYG*) was confirmed by Southern blot using probes for the *TbGT15* ORF and 3′ UTR (Fig. 1*B*). To allow for a tetracycline-inducible re-expression of the gene, an ectopic copy of *TbGT15* was introduced into the rRNA locus of the null mutant background using the pLEW100 vector (31). Clones were selected on phleomycin, and the creation of this conditional null mutant (Δ*TbGT15*^Ti^/Δ*TbGT15*::*PAC*/Δ*TbGT15*::*HYG*) was confirmed by RT-PCR (Fig. 1*C*).

**FIGURE 2.**
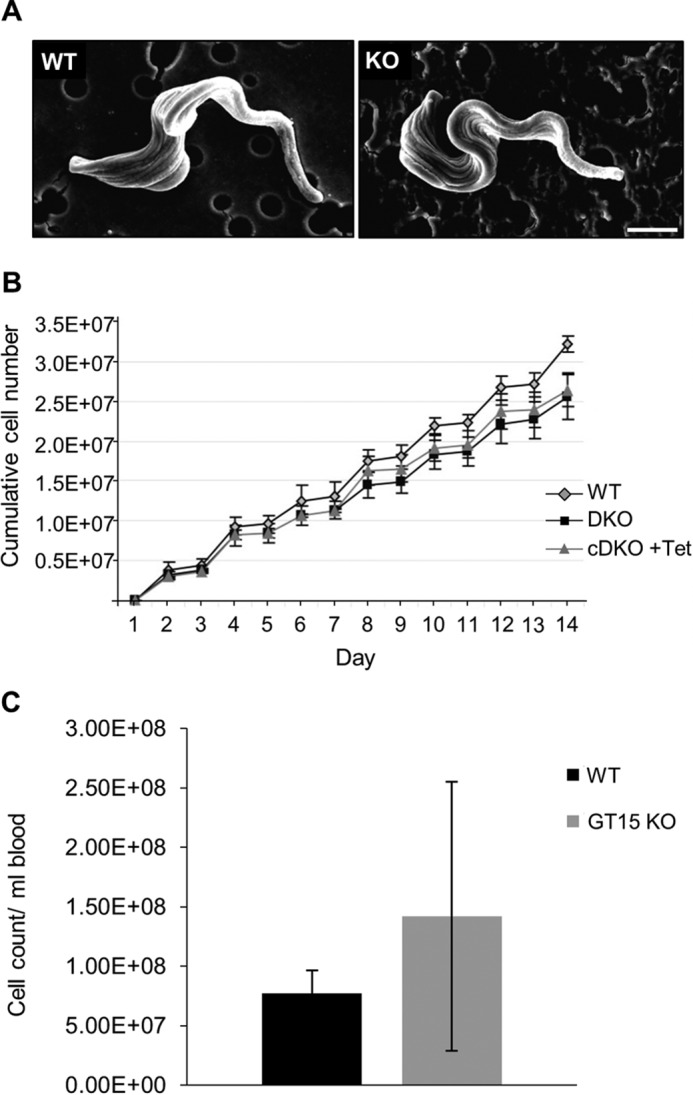
**Absence of *TbGT15* does not affect bloodstream form trypanosome morphology and growth.** *A*, scanning electron micrographs of representative WT (*left panel*) and *TbGT15* null mutant cells (*right panel*); *scale bar,* 2 μm. *B*, growth curves for bloodstream form *T. brucei* wild-type cells (*WT*; *gray diamonds*), *TbGT15* null mutant cells (*TbGT15*DKO; *black squares*), and *TbGT15* conditional null mutant cells grown under permissive conditions (*TbGT15*cDKO; *gray triangles*). Cell counts were taken daily in triplicate; *error bars* indicate one S.D. of the mean. *C*, infectivity of wild-type and *TbGT15* null mutant bloodstream from parasites to mice. Mice were infected with 5 × 10^5^ cells of WT (*black*) or *TbGT15* null mutants (*gray*), and the number of parasites per ml blood was counted 3 days post-infection. No difference in infectivity was observed.

No morphological differences between the WT and *TbGT15* null mutant parasites could be ascertained by light microscopy or by scanning electron microscopy (Fig. 2*A*). Compared with WT cells, the *TbGT15* null mutant parasites exhibited slightly slower growth kinetics *in vitro,* and this mild growth phenotype was partially reversed in *TbGT15* conditional null cells grown under permissive conditions (Fig. 2*B*). In addition, no difference in its ability to infect mice could be detected for the *TbGT15* null mutant (Fig. 2*C*). From this we can conclude that *TbGT15* is a non-essential gene in *T. brucei* bloodstream form cells.

##### Characterization of VSG from WT and TbGT15 Null Mutant Parasites

VSG221 from WT cells is heterogeneously glycosylated, containing a highly galactosylated GPI anchor (5), one oligomannose *N-*glycan at Asn-428 (Man_5_-_9_GlcNAc_2_), as well as small biantennary structures ranging from Man_3_GlcNAc_2_ to Gal_2_GlcNAc_2_Man_3_GlcNAc_2_ at Asn-296 (8, 16). VSG can be isolated in its sVSG form by hypotonic lysis, which results in its release by of endogenous GPI-specific phospholipase C (44).

**FIGURE 3.**
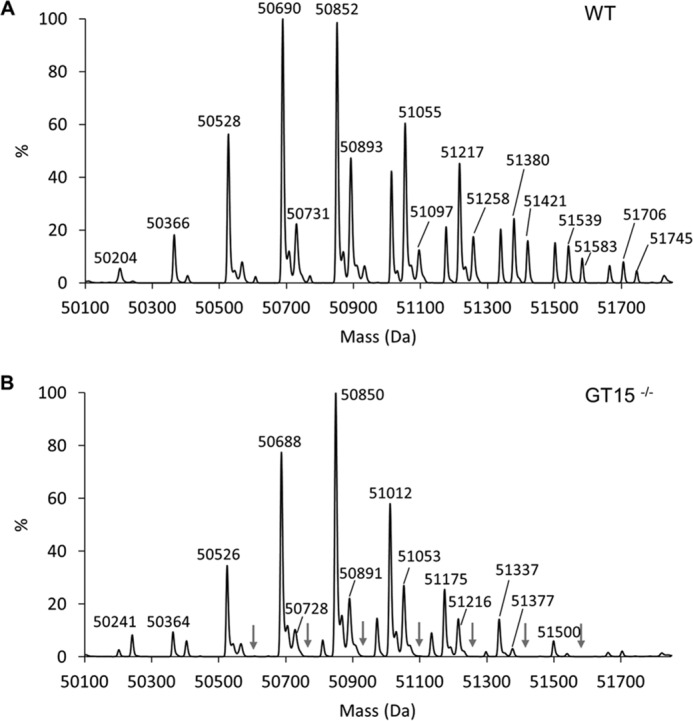
**sVSG221 glycoform analysis by ES-MS.** Samples of intact sVSG of WT (*A*) and *TbGT15* null mutant trypanosomes (*B*) were analyzed by positive-ion ES-MS, and the deconvolved spectra of the various isobaric glycoforms were generated (the compositions of these glycoforms are given in Table 1). Significant differences in the sVSG glycosylation patterns are indicated by *arrows* in *B*.

To assess differences in the glycosylation phenotype, intact sVSGs from WT and *TbGT15* null mutant parasites were analyzed by ES-MS in positive-ion mode (Fig. 3). VSG molecules containing a total of four or five GlcNAc residues were present at similar levels in both genotypes, but glycoforms with six GlcNAc residues were completely absent in the *TbGT15* null mutant (see *arrows* in Fig. 3*B* and Table 1). Bearing in mind that four GlcNAc residues are necessary for the composition of the two *N-*glycan *N*-acetylchitobiose core structures, the lack of VSG glycoforms containing six GlcNAc residues strongly indicates a deficiency in biantennary complex *N*-glycans at Asn-296. From this we can conclude that the mutant cells are unable to express complex *N*-glycans and that TbGT15 is involved in their biosynthesis.

**TABLE 1:** **Isobaric glycoforms of sVSG221 identified by ES-MS** The molecular masses of different glycoforms of sVSG221 were calculated according to the indicated compositions (the theoretical mass of the assigned VSG composition is shown in parentheses). The relative abundances of those glycoforms observed in Fig. 3 for sVSG preparations from sVSG of WT cells and *TbGT15* null mutant cells are indicated by −, trace, +, ++, and +++ scores. NA means not applicable.

Mass (in Da) WT/*TbGT15* null mutant (theoretical)	Protein*^a^*	GlcN-Ino-cP*^b^*	EtNP	GlcNAc	Man +Gal	WT	*TbGT15* null mutant
50,204/50,202 (50,194)	1	1	1	4	16	+*^c^*	Traces
50,241/50,242 (50,235)	1	1	1	5	17	Traces	+
50,366/50,364 (50,356)	1	1	1	4	17	+	+
50,405/50,404 (50,397)	1	1	1	5	16	Traces	+
50,528/50,526 (50,518)	1	1	1	4	18	+++	++
50,568/50,566 (50,559)	1	1	1	5	17	+	Traces
50,608/NA (50,600)	1	1	1	6	16	Traces	−
50,690/50,688 (50,680)	1	1	1	4	19	+++	+++
50,731/50,728 (50,721)	1	1	1	5	18	+	+
50,770/NA (50,762)	1	1	1	6	17	Traces	−
50,852/50,850 (50,842)*^d^*	1	1	1	4	20	+++	+++
50,893/50,891 (50,883)	1	1	1	5	19	++	+
50,933/NA (50,924)	1	1	1	6	18	+	−
51,014/51,012 (51,004)	1	1	1	4	21	++	+++
51,055/51,053 (51,045)	1	1	1	5	20	+++	+
51,097/NA (51,086)	1	1	1	6	19	+	−
51,177/51,175 (51,166)	1	1	1	4	22	+	+
51,217/51,216 (51,207)	1	1	1	5	21	++	+
51,258/NA (51,248)	1	1	1	6	20	+	−
51,340/51,337 (51,328)	1	1	1	4	23	+	+
51,380/51,377 (51,369)	1	1	1	5	22	+	Traces
51,421/NA (51,410)	1	1	1	6	21	+	−
51,502/51500 (51,490)	1	1	1	4	24	+	+
51,542/51,539 (51,531)	1	1	1	5	23	+	Traces
51,583/NA (51,572)	1	1	1	6	22	+	−
51,665/51,662 (51,652)	1	1	1	4	25	+	Traces
51,706/51,704 (51,693)	1	1	1	5	24	+	Traces
51,745/NA (51,734)	1	1	1	6	23	Traces	−

*^a^* Protein mass is based on the amino acid sequences of the VSG221 precursor (accession no. P26332) minus residues 1–27 (signal peptide) and 460–476 (GPI attachment signal peptide) and allows for four disulfide bonds (mass = 46,284 Da).

*^b^* Components specific to the GPI anchor and common to all glycoforms: GlcN-Ino-cP, glucosamine-α1–6-*myo*-inositol-1,2 cyclic phosphate; EtNP, ethanolamine phosphate.

*^c^* Maximum entropy deconvolved spectra are only semi-quantitative; an indication of relative abundance of the isobaric glycoforms is given based on peak height.

*^d^* The most abundant glycoform of WT sVSG221 is expected to contain a GPI anchor of composition of Man_3_Gal_5_ (5), a C-terminal *N*-linked glycan of Man_9_GlcNAc_2_, and an internal *N*-linked glycan of Man_3_GlcNAc_2_ (8) (*i.e*. GlcNAc = 4, and Man = 20).

##### In Vitro Functional Activity Assay

**FIGURE 4.**
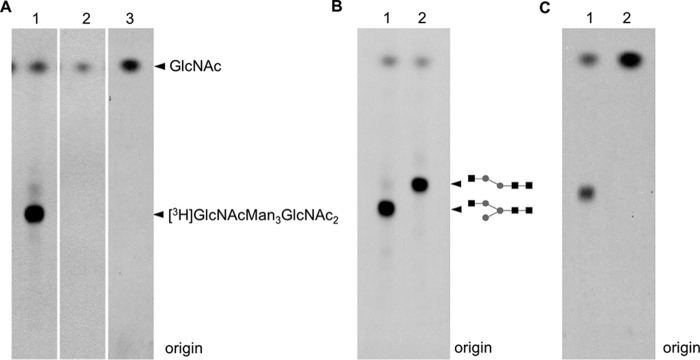
**TbGT15 *in vitro* functional activity assay.** Fluorographs of HPTLC plates showing the products of UDP-[^3^H]GlcNAc and anti-HA-conjugated magnetic bead immunoprecipitates from *T. brucei* expressing TbGT15-HA_3_ incubated with Manα1–3(Manα1–6)Manβ1–4GlcNAcβ1–4GlcNAcor Manα1–6(Manα1–3)Man (*lane 1* or *3*) (*A*) are shown. As a negative control, anti-HA-conjugated magnetic beads incubated with lysates from cells not expressing TbGT15-HA_3_ were used with Manα1–3(Manα1–6)Manβ1–4GlcNAcβ1–4GlcNAc (*lane 2*). *B*, [^3^H]GlcNAcMan_3_GlcNAc_2_ reaction product before (*lane 1*) and after (*lane 2*) treatment with α1–2,3 mannosidase. *C*, [^3^H]GlcNAcMan_3_GlcNAc_2_ reaction product before (*lane 1*) and after (*lane 2*) treatment with β-*N*-acetylglucosaminidase.

To verify that *TbGT15* encodes a glycosyltransferase that is directly involved in the biosynthesis of hybrid or complex *N*-glycans, we performed an *in vitro* assay for enzymatic activity as described previously (1). Briefly, full-length *TbGT15* fused to a C-terminal 3× HA epitope tag was expressed in *T. brucei* bloodstream form cells and immunoprecipitated using anti-HA magnetic beads. The protein was incubated with Manα1–6(Manα1–3)Manβ1-4GlcNAcβ1–4GlcNAc as acceptor substrate and tritium-labeled UDP-[^3^H]GlcNAc as donor substrate. Following desalting and removal of excess UDP-[^3^H]GlcNAc donor by mixed-bed ion exchange, aliquots were separated by thin layer chromatography (TLC) and analyzed by fluorography. Although a control immunoprecipitation with lysate from WT cells did not result in any [^3^H]GlcNAc incorporation (Fig. 4*A, lane 2*), the sample containing TbGT15-HA_3_ showed a strong signal of tritium-labeled reaction product (Fig. 4*A, lane 1*). This demonstrates that TbGT15 is able to transfer GlcNAc to biantennary Manα1–6(Manα1–3)Manβ1–4GlcNAcβ1–4GlcNAc core structures. It is noteworthy that the shortened substrate Manα1–6(Manα1–3)Man lacking the chitobiose core was not used as an UDP-GlcNAc acceptor by TbG15 (Fig. 4*A*, *lane 3*).

To reveal whether GlcNAc is transferred to the Manα1–3 or Manα1–6 arm of Manα1–6(Manα1–3)Manβ1–4GlcNAcβ1-4GlcNAc, the reaction product was treated with α1–2,3-mannosidase. As seen in the subsequent TLC/autofluorography analysis, mannosidase treatment caused an increased mobility of the reaction product (compare *R_f_* values in Fig. 4*B*). This suggests that the 3-Man arm was not modified by TbGT15, leaving it susceptible to exoglycosidase cleavage, and allows us to conclude that the transferred GlcNAc residue is attached to the 6-Man arm of the Manα1–6(Manα1–3)Manβ1-4GlcNAcβ1–4GlcNAc core structure. The anomeric configuration of the newly formed linkage was determined by β-*N*-acetylglucosaminidase digestion of the reaction product. In the following TLC/autofluorography analysis, the band of tritium-labeled GlcNAcMan_3_GlcNAc_2_ disappeared, although the amount of free [^3^H]GlcNAc increased, demonstrating a β-configuration (Fig. 4*C*).

**FIGURE 5.**
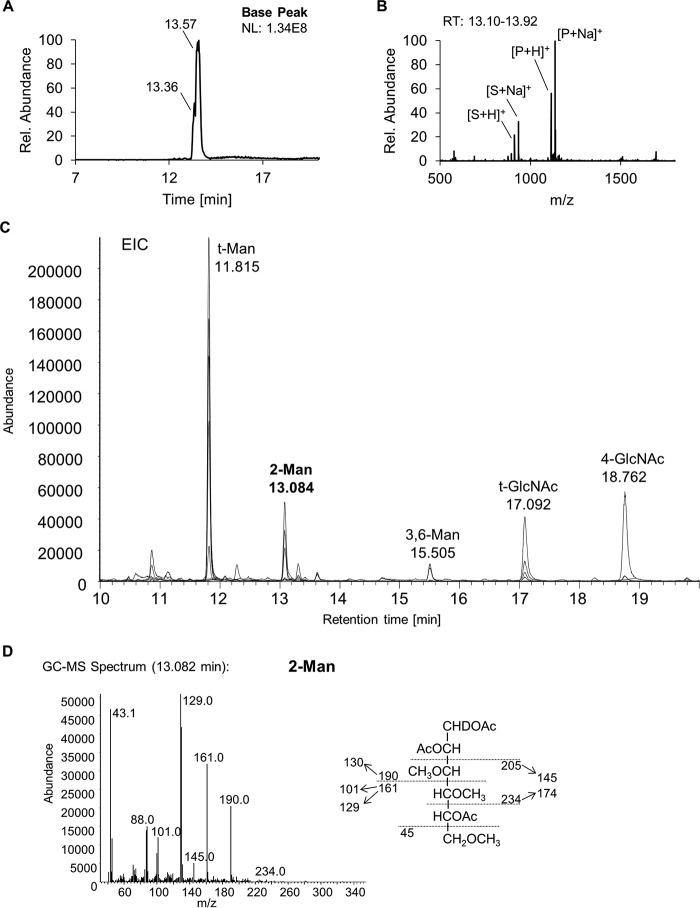
**Mass spectrometry analysis of TbGT15 reaction product.** HILIC-MS of *in vitro* assay reaction. *A*, ion chromatogram showing substrate HexNAc_2_Hex_3_ (eluting at 13.36 min) and reaction product HexNAc_3_Hex_3_ (eluting at 13.57 min). The data are adjusted such that 100% relative abundance corresponds to the normalization level (*NL*) of 1.34E8 ion counts. *B*, mass spectrum of HexNAc_2_Hex_3_ (substrate, *S*) and HexNAc_3_Hex_3_ (product, *P*) in protonated and sodiated form ([S + H]^+^ = 911.51; [S + Na]^+^ = 933.43; [P + H]^+^ = 1114.58; and [P + Na]^+^ = 1136.55). *C*, GC-MS extracted ion chromatogram of ions (*m/z* 102, 118, 129, 117, 161, and 190) characteristic for PMAA derivatives obtained after permethylation, acid hydrolysis, sodium borodeuteride reduction, and peracetylation of the TbGT15 reaction product. *EIC*, extracted ion chromatogram. *D*, spectrum of the 1,2,5-tri-*O-*acetyl-(1-deutero)-3,4,6-tri-*O-*methyl-mannitol PMAA derived from 2-*O-*substituted mannose, with characteristic fragment ion assignments.

For further characterization of the reaction product, the assay was performed using non-radioactive UDP-GlcNAc. First, HILIC-MS was performed to identify the HexNAc_3_Hex_3_ reaction product (Fig. 5, *A* and *B*). A subsequent methylation linkage analysis on the reaction sample by GC-MS demonstrated the presence of 1,2,5-tri-*O-*acetyl-(1-deutero)-3,4,6-tri-*O-*methyl-mannitol, originating from 2-*O-*substitued mannose, which reveals that TbGT15 transfers GlcNAc in a 1–2 linkage to one of the non-reducing terminal mannose residues of the trimannosyl core (Fig. 5, *C* and *D*).

Taken together, these data show that TbGT15 is the glycosyltransferase responsible for the transfer of β1–2-linked GlcNAc to the α1–6-linked α-d-mannopyranosyl residue of Manα1–6(Manα1–3)Manβ1–4GlcNAcβ1–4GlcNAc and can therefore be termed an *N*-acetylglucosaminyltransferase type II or TbGnTII.

##### N-Glycosylation Phenotype of Bloodstream Form TbGT15 Mutant Parasites

**FIGURE 6.**
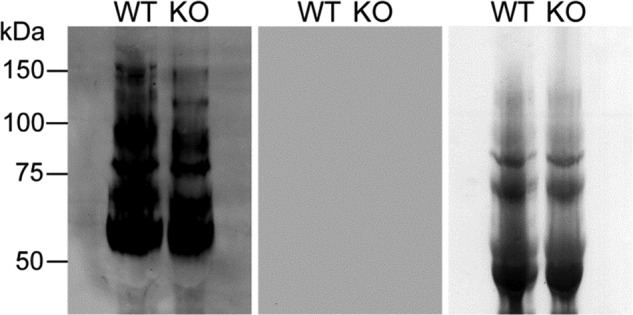
**Lectin blotting of VSG-depleted glycoproteins.** Lysates of washed WT or *TbGT15* null mutant (KO) trypanosome cell ghosts were subjected to SDS-PAGE and transferred to nitrocellulose membrane. The membrane was incubated with ricin (*left panel*) or, as a binding specificity control, with ricin that was pre-incubating with 10 mg/ml each of galactose and lactose (*middle panel*). Equal loading and transfer are demonstrated by Ponceau S staining (*right panel*).

To investigate the effect of TbGT15 on the glycosylation of other proteins than VSG, total glycoproteins were extracted with SDS/urea from VSG-depleted trypanosome ghosts and analyzed by lectin blotting. As reported previously for WT *T. brucei*, ricin (RCA-120), a lectin that predominantly binds to non-reducing terminal β-galactose residues, showed strong binding to a series of glycoproteins running between 60 and 150 kDa (Fig. 6, *1st lane*). Ricin binding to glycoproteins extracted from the *TbGT15* null mutants was slightly reduced, and the apparent molecular mass of all signals was marginally smaller compared with WT signals (Fig. 6, *2nd lane*). Albeit subtle, these changes in blotting pattern suggest an alteration in the synthesis of the large poly-LacNAc-containing glycans of the high molecular weight invariant glycoproteins (19).

To draw structural conclusions, we decided to analyze the ricin-binding glycoprotein fraction by methylation linkage as described previously (19). Briefly, WT and *TbGT15* null mutant cells were isolated, depleted of VSGs, solubilized in SDS/urea, and glycoproteins were purified by ricin affinity chromatography. *N*-Linked glycans were released by peptide:*N*-glycosidase F and further fractionated by Bio-Gel P-4 gel filtration, resulting in two main fractions as follows: one containing the relatively small mannose-rich *N*-glycans, and a Gal/GlcNAc-rich high molecular mass fraction eluting at the void volume of the Bio-Gel P-4 column (the total poly-LacNAc fraction). Aliquots of these fractions were subjected to neutral monosaccharide composition analysis by GC-MS. The molar ratios of Gal/Man in the total poly-LacNAc fraction for WT (19) and *TbGT15* null mutant parasites were found to be similar, 14.5:1 and 12.4:1, respectively.

**FIGURE 7.**
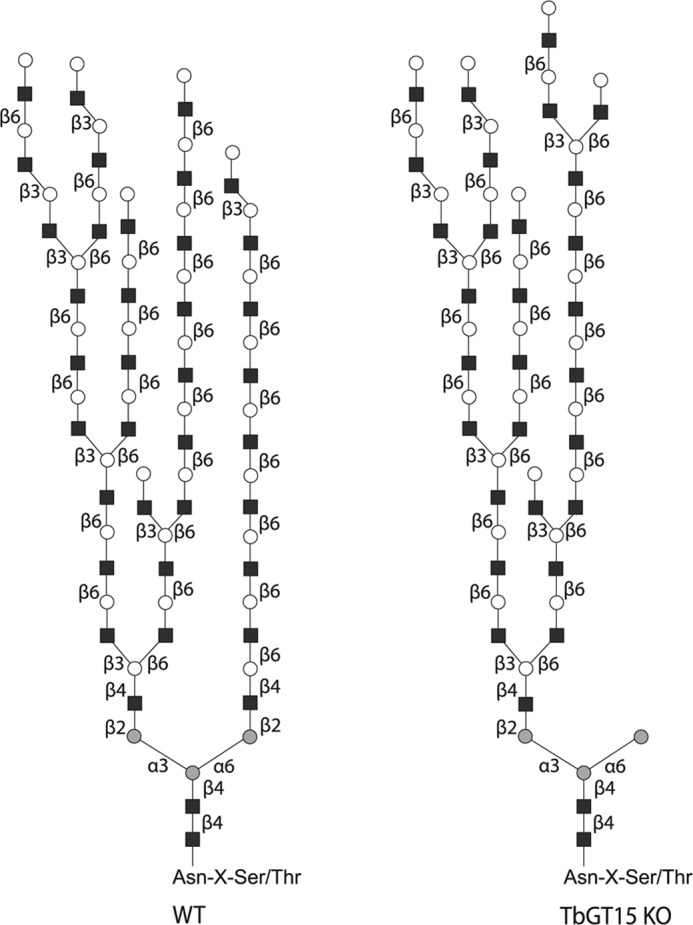
**Proposed scheme for poly-LacNAc-containing *N-*glycans of bloodstream form trypanosomes.** The data presented here and in Ref. 1 are consistent with the model shown here whereby in the wild-type bloodstream form of *T. brucei* the majority of large complex *N*-glycans carry highly branched poly-LacNAc chains on the Manα1–3 arm and predominantly linear poly-LacNAc chains on the Manα1–6 arm. In the *TbGT15* null mutant, all elaboration of the Manα1–6 arm is absent and partly compensated by an increase in the branched poly-LacNAc elaboration of the Manα1–3 arm. Note that the relative positions of branch points shown are arbitrary.

Subsequent GC-MS methylation linkage analysis of the total poly-LacNAc fraction revealed structural similarities but also some quantitative differences (Table 2). Thus, both WT and *TbGT15* null total poly-LacNAc glycans contain 2-*O-*substituted Man and 3,6-di-*O-*substituted Man, consistent with a conventional core structure of R-2Manα1–6(R′-2Manα1–3)Manβ1–4GlcNAcβ1–4GlcNAc in all structures. Furthermore, both samples contained significant amounts of 4-*O-*substituted GlcNAc, indicating the presence of multiple LacNAc repeats. However, although the numbers of terminal Gal residues were similar, there was a significant decrease in 6-*O-*substituted Gal and 3-*O-*substituted Gal residues and a concomitant increase in 3,6-di-*O-*substituted-Gal residues in the *TbGT15* null mutant glycans. Because TbGT15 has a GnTII-type activity that initiates elaboration of the Manα1–6 arm of Manα1–6(Manα1–3)Manβ1–4GlcNAcβ1-4GlcNAc core, these data are consistent with a model where linear poly-LacNAc chains predominate on the Manα1–6 arm, and the Manα1–3 arm is occupied by both linear and branched poly-LacNAc units (Fig. 7). The increase in the number of 3,6-di-*O-*substituted Gal residues in the *TbGT15* mutant glycans further suggests that deletion of the entire (predominantly linear) poly-LacNAc chain attached to the Manα1–6 arm is, to some extent, compensated by further elaboration of the (linear and branched) poly-LacNAc chain attached to the Manα1–6 arm.

**TABLE 2:** **Quantitative GC-MS methylation linkage analysis of the total poly-LacNAc fraction** The total poly-LacNAc fraction was permethylated, hydrolyzed, reduced, and acetylated for GC-MS analysis. The resulting PMAA derivatives were identified by retention time and electron impact mass spectra. Quantification was accomplished by integration of the total ion current using molar relative response factors deduced empirically from authentic standards, as described under “Experimental Procedures.”

PMAA derivative	Origin	WT*^a^*	*TbGT15* null mutant*^a^*
2,4-Di-*O-*methyl-1,3,5,6-tetra-*O-*acetyl-1-[^2^H]mannitol	3,6-Di-*O*-substituted Man	1.0	1.0
3,4,6-Tri-*O-*methyl-1,2,5-tri-*O-*acetyl-1-[^2^H]mannitol	2-*O*-Substituted Man	2.3	2.3
2,3,4,6-Tetra-*O*-methyl-1,5-di-*O*-acetyl-1-[^2^H]galactitol	Terminal Gal	4.9	5.0
2,4,6-Tri-*O*-methyl-1,3,5-tri-*O*-acetyl-1-[^2^H]galactitol	3-*O*-Substituted Gal	2.2	1.7 (down 23%)
2,3,4-Tri-*O*-methyl-1,5,6-tri-*O*-acetyl-1-[^2^H]galactitol	6*-O*-Substituted Gal	23.4	15.9 (down 32%)
2,4-Di-*O*-methyl-1,3,5,6-tetra-*O*-acetyl-1-[^2^H]galactitol	3,6-Di-*O*-substituted Gal	3.6	5.0 (up 39%)
3,6-Di-*O*-methyl-1,4,5-tri-*O*-acetyl-2-*N*-methylacetamido-1-[^2^H]glucosaminitol	4-*O*-substituted GlcNAc	24.6*^b^*	16.6*^b^*

*^a^* Molar quantities relative to 3,6-di-*O*-substituted Man (one per glycan) are shown.

*^b^* Values for *N*-acetylglucosamine derivatives are less reliable than for hexoses.

## Discussion

We have cloned the gene that encodes UDP-GlcNAc:α1-6-d-mannoside-β1–2-*N*-acetylglucosaminyltransferase II in *T. brucei*, an enzyme that catalyzes an essential step on the route to complex *N*-glycans. In human patients with carbohydrate-deficient glycoprotein syndrome type II, congenital deficiency in GnTII activity is associated with severe psychomotor retardation and other multisystemic abnormalities (45, 46). In a mouse model with a homozygous null mutation in the gene encoding GnTII (*Mgat2*^−/−^), 99% of newborns die during the first postnatal week (47). These developmental defects highlight the importance of complex *N*-glycans in intercellular communication and signaling in multicellular organisms.

The significance of complex *N*-glycans in the unicellular protozoan *T. brucei* is less well understood. In the bloodstream form, the parasite expresses both conventional biantennary complex *N*-glycans and unique highly extended and branched poly-*N*-acetyllactosamine-containing complex *N*-glycans (8, 19, 48, 49). However, the *T. brucei* genome contains no obvious homologues of the canonical *GnTI* and *GnTII* genes that encode the β1–2GlcNAc transferases usually responsible for the initiation of complex *N*-glycans. In a previous study, we identified and characterized TbGnTI, the enzyme responsible for the transfer β1–2GlcNAc to the Manα1–3 arm of *N*-glycan core structures (1). TbGnTI showed unusual activity in that it acts on biantennary Man_3_GlcNAc_2_ instead of triantennary Man_5_GlcNAc_2_, the preferred acceptor substrate for vertebrate GnTI activities (50). Remarkably, the *TbGnTI* gene is highly divergent from the canonical *GnTI* gene family and, despite the fact that TbGnTI catalyzes a β1–2 linkage, it belongs to the so-called β3-glycosyltransferase superfamily (1, 26).

**FIGURE 8.**
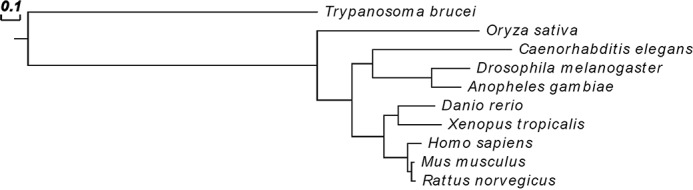
**Phylogenetic tree of GnTII amino acid sequences from different species.** Amino acid sequences were aligned using the COBALT constraint-based multiple alignment program. GnTII, *Oryza sativa* (NP_001048400.2); *Caenorhabditis elegans* (NP_505864.1); *Drosophila melanogaster* (NP_651763.4);*Anopheles gambiae* (XP_313681.5); *Danio rerio* (NP_001077344.1); *Xenopus tropicalis* (NP_001006759.1); *Homo sapiens* (NP_002399.1); *Mus musculus* (NP_666147.1); and *Rattus norvegicus* (NP_446056.1). The evolutionary distance is represented by the length of the *horizontal lines*.

In this study, a reverse-genetics approach in *T. brucei* bloodstream form cells indicated that the deletion of *TbGT15* (another trypanosome β3-glycosyltransferase superfamily member) is accompanied by the absence of complex *N*-glycans, as well as alterations in the biosynthesis of the giant poly-LacNAc-containing glycans. Using a direct enzymatic assay and comprehensive product analysis, we could show that purified TbGT15 catalyzes the conversion of Manα1–6(Manα1–3)Manβ1–4GlcNAcβ1–4GlcNAc to GlcNAcβ1–2Manα1–6(Manα1–3)Manβ1–4GlcNAcβ1–4GlcNAc. We have therefore renamed TbGT15 to TbGnTII. The conversion of Man_3_GlcNAc_2_ demonstrates that TbGnTII works independently from prior TbGnTI action, which is in contrast to canonical GnTIIs that only use substrates after modification by GnTI, *i.e.* Manα1–6(GlcNAcβ1–2Manα1–3)Manβ1–4GlcNAcβ1-4GlcNAc (51, 52). This unusual acceptor specificity of the trypanosome enzyme was already indicated by previous data, which show the presence of “pseudohybrid” *N*-glycans in the absence of TbGnTI (1) and highlights the divergent nature of TbGnTII. A phylogram based on a multiple sequence alignment of TbGT15 and GnTII enzymes of other species is shown in Fig. 8. The GnTIIs of multicellular organisms belong the CAZy (carbohydrate-active enzymes) GT family 13 (39) and are all closely related, with nearly 90% identity between the human and mouse enzymes. In contrast, TbGnTII is a member of the CAZy GT31 family (26) and shares only 9% identity with the human sequence at the amino acid level. Interestingly, although human GnTI and GnTII proteins have only a low level of amino acid sequence homology between them (22%), the TbGnTI and TbGnTII enzymes share 42% identity. This is consistent with the closer functional similarity of the trypanosome enzymes, both of which work on the same acceptor substrate (Man_3_GlcNAc_2_, although only the latter requires the *N*-acetylchitobiose core), whereas the canonical GnTI and GnTII enzymes work on triantennary Man_5_GlcNAc_2_ and Manα1–6(GlcNAcβ1-2Manα1–3)Manβ1–4GlcNAcβ1–4GlcNAc, respectively.

Methylation linkage analysis of the poly-LacNAc *N*-glycans of the *TbGT15* null mutant showed a reduction in 6-*O-*substituted Gal and 3-*O-*substituted Gal but an increase in 3,6-*O-*substituted Gal. This allows us to augment our model of the parasites' complex *N*-glycans and propose that the Manα1–6 arm is normally occupied by predominantly linear poly-LacNAc repeats and the Manα1–3 arm by branched as well as linear poly-LacNAc repeats (Fig. 7).

*T. brucei* has an unusual dual *N*-glycosylation mechanism with two paralogous oligosaccharyltransferases, TbSTT3A and TbSTT3B, that transfer biantennary Man_5_GlcNAc_2_ and triantennary Man_9_GlcNAc_2_, respectively, in a site-specific manner (15, 16). Because of the absence of Golgi α-mannosidase II in the parasite, triantennary structures cannot be processed to complex *N-*glycans, rendering biantennary Man_5_GlcNAc_2_ transferred by STT3A the only route to paucimannose and complex structures. Furthermore, the inability of TbGnTI to act on triantennary Man_5_GlcNAc_2_ (1) also means that biantennary Man_5_GlcNAc_2_ transferred bySTT3A is the only possible route to pseudohybrid *N*-glycans (*i.e.* those with only one arm of the trimannosyl-core modified by GlcNAc ± additional sugars). RNAi knockdown of TbSTT3A showed that cells are viable in culture but not in mice (15). Interestingly, the deletion of *TbGnTI* (*TbGT11*) has no effect on *in vitro* growth rate, and the infectivity to mice was indistinguishable from wild type (1). This suggests that the presence of pseudohybrid *N*-glycans with glycan extensions to the 6-arm alone are sufficient to compensate for the loss of complex *N*-glycans. Here, the *in vitro* and *in vivo* viability of the *TbGnTII* null mutant shows that the reverse is true, in that the presence of hybrid structures with extensions to the 3-arm alone compensates for the loss of complex *N*-glycans. However, despite extensive attempts, a double knock-out lacking both *TbGnTI* and *TbGnTII* genes could not be generated in our hands, suggesting that extension of one or other of the arms of the *N*-glycan trimannosyl-core is essential for the growth and infectivity of bloodstream form of *T. brucei.*

## Author Contributions

M. D. and M. A. J. F. designed the research and wrote the paper. M. D. and F. G. performed and analyzed experiments. M. L. S. G. performed mouse infectivity studies. A. M. and L. I. assisted in the creation of the TbGT15 mutants and the isolation and GC-MS analysis of the TbGT15 mutant glycans. All authors reviewed the results and approved the final version of the manuscript.

## Footnotes

8 The abbreviations used are:

GPI: glycosylphosphatidylinositol
VSG: variant surface glycoprotein
sVSG: soluble form VSG
poly-LacNAc: poly-*N*-acetyllactosamine
GT: glycosyltransferase
GnT: GlcNAc transferase
PAC: puromycin acetyltransferase
HYG: hygromycin phosphotransferase
Hex: hexose
HexNAc: *N*-acetylhexosamine
HILIC: hydrophilic interaction liquid chromatography
PMAA: partially methylated alditol acetate
TLCK: tosyllysine chloromethyl ketone hydrochloride
CAZy: carbohydrate-active enzyme
bis-Tris: 2-[bis(2-hydroxyethyl)amino]-2-(hydroxymethyl)propane-1,3-diol.
